# Hydroxysafflor Yellow A (HSYA) from Flowers of *Carthamus tinctorius* L. and Its Vasodilatation Effects on Pulmonary Artery

**DOI:** 10.3390/molecules171214918

**Published:** 2012-12-13

**Authors:** Yuhua Bai, Ping Lu, Chenghua Han, Chunyue Yu, Minggang Chen, Fa He, Dan Yi, Lijun Wu

**Affiliations:** 1 College of Pharmacy, Harbin Medical University, Harbin 150081, Heilongjiang, China; 2 College of Pharmacy, Harbin Medical University-Daqing, Daqing 163319, Heilongjiang, China

**Keywords:** HSYA, *Carthamus tinctorius* L., pulmonary arteries, vasodilatation

## Abstract

Flowers of *Carthamus tinctorius* L. are traditionally used in China to treat cerebrovascular and cardiovascular diseases. Hydroxysafflor yellow A (HSYA), the main constituent of *Carthamus tinctorius* L. flowers, is known for its multiple biological activities. In the present study, HSYA was isolated from *Carthamus tinctorius* L. flowers by a macroporous resin adsorption chromatography method coupled with a Waters high-throughput auto-purification system and it’s vasodilatation effects on pulmonary artery (PA) were explored by an assay of tension study on rat pulmonary artery (PA) rings. Results suggest that HSYA possesses vascular relaxation effects on rat PA by activating the KV channel in pulmonary vascular smooth muscle cells (PVSMCs).

## 1. Introduction

Pulmonary arterial hypertension (PAH) is a progressive disease characterized by increased pulmonary vascular resistance, leading to chronic elevation in pulmonary arterial pressure resulting from restricted flow through the pulmonary arterial circulation system. These pathobiological features typically lead to right-side heart failure and premature death [1,2]. Although available treatments can improve prognosis, this disease has been considered incurable and with poor survival [3,4]. Treatments for PAH have evolved in the last decade because of a greater understanding of the disease pathology, as well as increased availability of medications targeting known affected pathways, but application of these medications is constricted owing to their serious side effects, which are hard to overcome. For example, the side effects of endothelium receptor antagonists include potential for hepatotoxicity, anemia, and edema [5], those of phosphodiesterase inhibitors include headaches, epistaxis, flushing, and dyspepsia [6]; moreover, most prostanoids are pharmacologically unstable, and adverse effects of their subcutaneous administration include pain or erythema at the site. Other common side effects include headache, nausea, rash, and diarrhea [7]. The conventional therapies have never been proven to be beneficial in a randomized, prospective manner. Accordingly, new alternative treatments of PAH are currently being explored, especially those derived from herbal plants which may possess diverse bioactivities.

**Figure 1:**
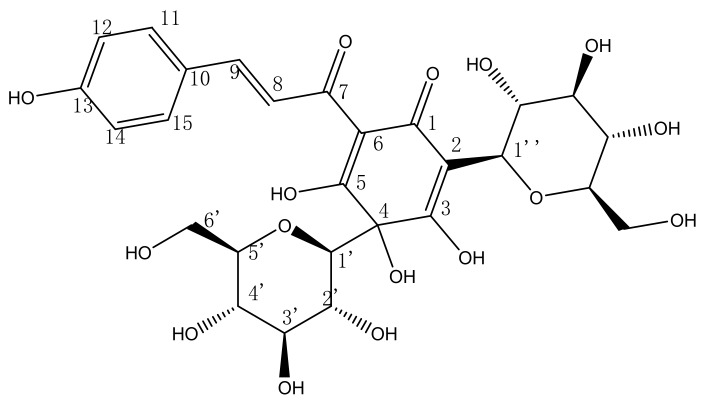
The chemical structure of HSYA.

*Carthamus tinctorius* L. is a kind of therophyte belonging to the Dicotyledoneae subclass, Compositae family [8]. Flowers of *Carthamus tinctorius* L. are used extensively in traditional Chinese medicine for the treatment of cerebrovascular and cardiovascular diseases [9,10,11]. 2,4-di-β-d-glucopyranosyl-3,4,5-trihydroxy-6-[(2*E*)-3-(4-hydroxyphenyl)-1-oxo-2-propenyl]-2,5-cyclohexadien-1-one (HSYA, Figure 1), the major bioactive compound of *Carthamus tinctorius* L., has been shown to antagonize platelet-activating factor receptor binding [12] and reduce myocardial infarct size [13]. Hypotensive and anti-thrombotic activities and inhibitory effects on platelet aggregation have also been reported [14,15]. It was also reported that HSYA significantly reduces blood pressure and heart rate [16], protects HUVECs (Human Endothelial Cells from Umbilical Cords) from hypoxia-induced injuries by inhibiting cell apoptosis and cell cycle [17]. Moreover, HSYA caused a concentration-dependent anti-contraction effects by KC l or PE in endothelium-intact and endothelium-denuded aortic rings [18]. However, there has been no report on the effects of *Carthamus tinctorius* L. and HSYA on pulmonary artery, so in the present study we explore the vasodilatation effects of HSYA on rat pulmonary artery and its potential mechanism.

## 2. Results and Discussion

### 2.1. HPLC Chromatography of F2 from Carthamus tinctorius *L.* Crude Extract and Separated HSYA

**Figure 2:**
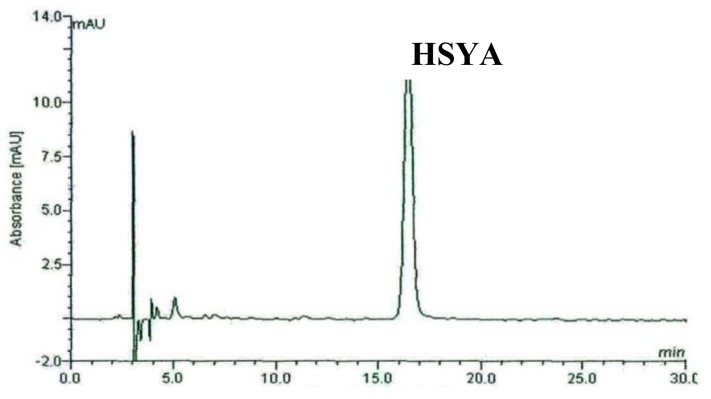
The HPLC chromatograph of the second fraction (F2) of crude extract from *Carthamus tinctorius* L. flowers obtained from a macroporous resin column. Conditions: column: ODS C_18_ (250 mm × 4.6 mm I.D., 5 μm); mobile phase: 0.2% formic acid in water (**A**), acetonitrile (**B**) and methanol (**C**) (A:B:C = 62:2:36) in isocratic elution mode for 30 min. The flow rate was 1 mL/min, aliquots of 10 µL were injected and the absorbance was recorded at 403 nm.

**Figure 3:**
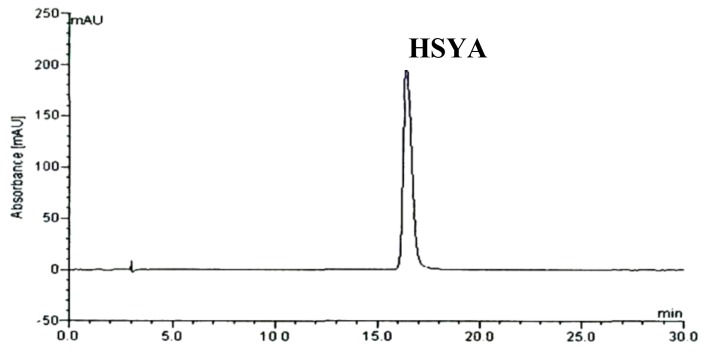
HPLC chromatograph of the separated HSYA. Conditions: same as Figure 2.

In order to get good resolution of adjacent peaks within a short analysis time, the optimum HPLC conditions were tested. In the beginning, various mixtures of water-methanol and acetonitrile-water were selected as mobile phase in the analysis of the fractions from *Carthamus* *tinctorius* L. separated by a D101 macroporous resin column, but the separation was not satisfactory. Thus 0.2% formic acid was added to ensure a better separation. The results indicated that when the methanol- formic acid in water (0.2%) and acetonitrile were used as mobile phase (water–acetonitrile–methanol = 62:2:36) in isocratic mode, major peaks showed a good separation. The HPLC chromatograph of the F2 is shown in Figure 2. HPLC chromatograph of separated HSYA obtained under the same HPLC conditions as the above is shown in Figure 3. The structure of the separated HSYA was further confirmed by IR, NMR and positive ESI-MS data.

### 2.2. Effects on PA Rings

#### 2.2.1. Effect of HSYA (10^−9^–10^−5^ M) on PE-Induced Vascular Constriction

**Figure 4:**
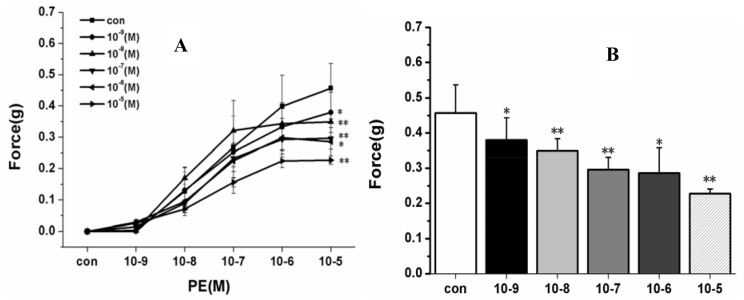
Vascular effect of HSYA on PE-induced constriction in rat PA rings with different concentration (10^−9^ M–10^−5^ M). * *p* < 0.05 compared with control; ** *p* < 0.01 compared with control.

To determine the vascular effects of HSYA on rat pulmonary artery rings, we tested different concentrations of HSYA (10^−9^–10^−5^ M). HSYA (10^−9^ M, 10^−8^ M, 10^−7^ M, 10^−6^ M, 10^−5^ M) was added to the bath and incubated for 30 min. After 30–40 min equilibration with a 0.3 g pre-load, the PA rings were then exposed to phenylephrine with a concentration gradient from 10^−9^ M–10^−5^ M and the data recorded. Results are shown in Figure 4A,B. 

#### 2.2.2. Effect on Endothelium-Intact PA Rings

**Figure 5:**
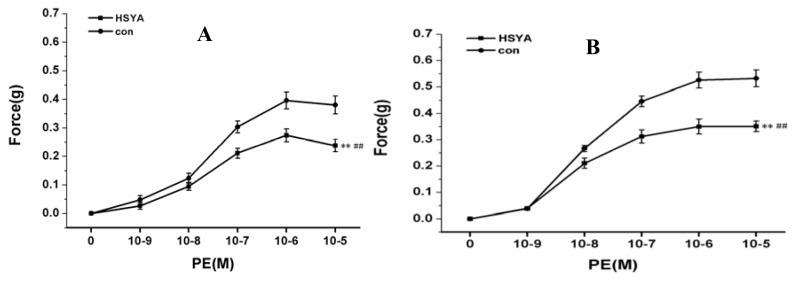
Vascular effect of HSYA on rat PA rings. (**A**) Vascular effect of HSYA on PE-induced vascular constriction in endothelium-intact rat PA rings; (**B**) Vascular effect of HSYA on PE-induced vascular constriction in endothelium-denuded rat PA rings. ** indicates the result has statistical significance (*p* < 0.01, compared with Normal). ## *p* < 0.01 compared with PE.

To determine the vascular effects of HSYA on rat pulmonary artery rings, endothelium-intact PA rings were studied in these experiments. As shown in Figure 5A, HSYA (10^−5^ M) had no effects on the rest tone of the rings. However, after 30 min incubation with HSYA and 30–40 min equilibration with a 0.3 g pre-load, the PA rings were then exposed to phenylephrine with a concentration gradient from 10^−9^ M–10^−5^ M. The exposure produced rapid contraction of these rings by 0.02–0.28 g and 0.05–0.4 g compared with control. The peak contraction was reached in 25 min and maintained for at least 30 min without evident decline. HSYA (10^−9^ M to 10^−5^ M) elicited a vasodilatation effect on the phenylephrine-induced restriction of artery rings (data was not shown). Basal tension of 0.3 g (passive force) was regarded as 100%. These results suggest that HSYA is effective at resisting PE-induced vascular constriction and this effect is HSYA concentration-dependent. 

#### 2.2.3. Effect on Endothelium-Denuded PA Rings

Using the same experimental procedure as above, vasorelaxing effects on endothelium-denuded PA rings were studied and the result was as shown in Figure 5B. The exposure produced a rapid contraction of these rings by 0.02–0.30 g and 0.03–0.50 g compared with control. Results indicate that HSYA is effective in resisting PE-induced vascular constriction and this effect is HSYA concentration-dependent. From the above experimental data, we can draw the conclusion that the vasorelaxing effect of HSYA has no relation with the PA endothelium.

#### 2.2.4. Effect on PA Rings after Blocking K^+^ Channels of PVSMCs

**Figure 6:**
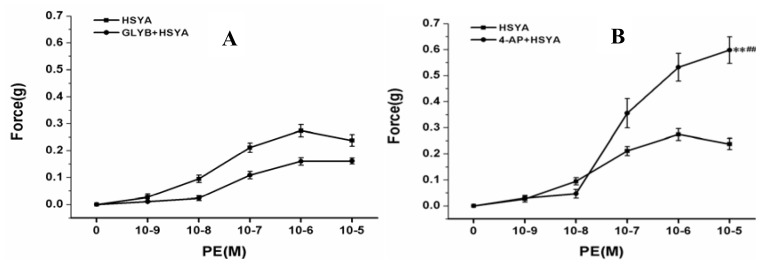

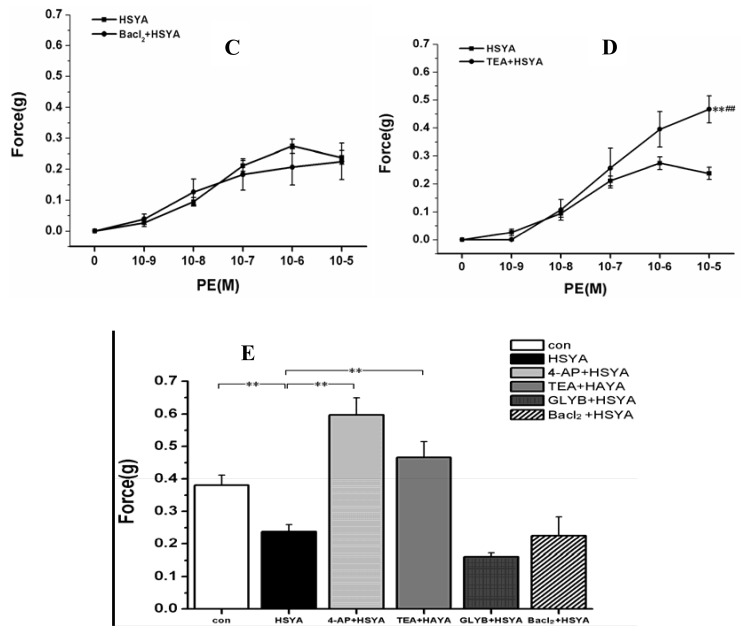
Vascular effect of HSYA after blocking K+ channels of PVSMCs. (**A**) Vascular effect of HSYA after pretreatment with GLYB; (**B**) Vascular effect of HSYA after pretreatment with 4-AP; (**C**) Vascular effect of HSYA after pretreatment with Bacl2; (**D**) Vascular effect of HSYA after pretreatment with TEA; (**E**) Bar graph shows the vascular effect of HSYA after blocking the different K+ channels of PVSMCs. ** indicates the result has statistical significance (*p* < 0.01, compared with Control). ## *p* < 0.01 compared with HSYA.

The experimental procedure was the same as in 2.2.1, but before adding HSYA, the different K^+^ channel blockers glibenclamide (GLYB, KATP channel blocker), 4-Aminopyridine (4-AP, K_v_ channel blocker), Bacl_2_ (KIR channel blocker) and tetraethylammonium (TEA, a non-selective kv channel blocker) were added and incubated for 30 min, respectively. As shown in Figure 6A–E, the results suggest that the vascular relaxation effect of HSYA is significantly dependent on the Kv channels of PVSMCs. 

## 3. Experimental

### 3.1. Apparatus

The separation instrument employed in the present study was a Waters 2545 high throughput preparation and purification system coupled with a UV detector (Waters Corporation, Milford, PA, USA), an ODS C18 column (250 mm × 19 mm I.D., 5 μm); The HPLC equipment was an Agilent 1200 HPLC system including a G1311A Quat-pump, a G1315D UV-vis photodiode array detector, a G1329A auto-sampler, a G1322A degasser and Angilent HPLC workstation (Agilent Technologies, Waldbronn, Germany). The nuclear magnetic resonance (NMR) spectra were recorded on a Bruker Avance 300 spectrometer (Bruker Company, karlsruhe, Germany). Mass spectra were recorded on a Waters LCT-Premier spectrometer (Waters Corporation, Milford, PA, USA) in ESI ionization mode (positive). FTIR-8400S(SHIMADZU CORPORATION, Kyoto, Japan). Tension data acquisition was facilitated by using the CODAS software (Shanghai Alcott Biotech Co, Ltd., Shanghai, China), which allows waveform playback and analysis. Each PA ring was mounted on a force transducer (ALC-MPA, Shanghai Alcbio Biology Technology Co., Ltd., Shanghai, China) and placed in a water-jacketed organ bath. The system allows simultaneous recordings from eight rings.

### 3.2. Reagents and Materials

All organic solvents used for the preparation of crude sample and HPLC separation were of analytic grade (Guangcheng Chemical Factory; Tianjin; China). Methanol and acetonitrile used for HPLC analysis was of chromatographic grade (Yuwang Chemical Factory; Yucheng, China). HPLC-grade water was purified using a Milli-Q system (Millipore, Bedford, MA, USA). Phenylephrine was obtained from Sigma Chemical Co. (St. Louis, MO, USA). All other reagents were from common commercial sources. Flowers of *Carthamus tinctorius* L. were purchased from China National Pharmaceutical Group Corporation (Beijing, China) and air dried. The plant was authenticated by professor Donghua Wei of Harbin Medical University; and identified by comparison to a fully registered specimen (voucher number: 20110905) following the Chinese National Pharmacopoeia; kept at the herbarium of pharmacy college of Harbin Medical University-Daqing. 

### 3.3. Plant Extract Preparation and Isolation of HSYA

The fresh flowers of *Carthamus tinctorius* L. (2 kg) were shade dried and powdered, then extracted three times at 60 °C for 30 min in distilled water (20 L). After removal of the solvent by evaporation under reduced pressure, the residue was dissolved in 10% ethanol (1,000 mL), then evaporated to dryness under vacuum to afford a residue (72.3 g), which was subjected to D101 macroporous resin column chromatography (200 g) using water-methanol step-gradient elution (100: 0–90: 10–80: 20–40: 60–0: 100, v/v) to yield five fractions F1–F5. The fraction F2 (2.8 g) was subjected to separation on a Waters 2545 high throughput preparation and purification system at 25 °C, using methanol–acetonitrile–formic 0.2% in water (36:2:62) as mobile phase in isocratic elution mode. The flow-rate of the mobile phase was 17 mL/min. the effluents were monitored at 403 nm by UV detector and collected automatically to get HSYA (1.32 g). Structure identification was performed by nuclear magnetic resonance, mass spectrometry and infrared spectroscopy. Orange needle-like crystals, mp 184.2 °C. IR (KBr, cm^−1^): 3384, 2928, 1625, 1605, 1519, 1426. HR-ESI-MS (*m/z*): 611.1450, (C_27_H_31_O_16_). ^1^H-NMR: (DMSO-d6, 300 MHz):18.13 (1H, s, 3-OH), 10.05 (1H, s, 5-OH), 7.75 (1H, d, *J* = 15 Hz, H-8), 7.52 (1H, d, *J* = 15 Hz, H-9), 7.55 (1H, d, H-11), 6.83 (2H, d, *J* = 9 Hz, 12, H-14), 7.42 (2H, d, *J* = 9 Hz, 11, H-15), 3.91 (1H, d, *J* = 9.5 Hz, H-l’), 3.54 (1H, d, H-2’), 2.50 (1H, m, H-4’), 3.80 (1H, m, H-6’), 4.23 (1H, d, *J* = 9.5Hz, H-l’’), 4.10 (1H, m, H-2’’), 3.18 (1H, m, H-3’’), 3.12 (1H, m, H-4’’), 3.42 (1H, d, H-6’’). ^13^C-NMR (DMSO-d_6_, 100 MHz): 190.3 (s, C-l), 105.3 (s, C-2), 195.6 (s, C-3), 87.0 (s, C-4), 181.0 (s, C-5), 90.2 (s, C-6), 183.7 (s, C-7), 119.4 (s, C-8), 144.0 (s, C-9), 128.7 (s, C-10), 130.0 (d, C-11, 15), 117.3 (d, C-12, 14), 157.8 (s, C-13), 87.2 (d, C-l’), 72.0 (d, C-2’), 77.0 (d, C-3’), 71.3 (d, C-4’), 80.5 (d, C-5’), 62.6 (t, C-6’), 74.7 (d, C-l’’), 67.5 (d, C-2), 79.5 (d, C-3’’), 70.1 (d, C-4’’), 81.3(d, C-5’’), 63.7 (t, C-6’’). The ^1^H-NMR and ^13^C-NMR data were compared with the data given in literature [19], and the product thus confirmed to be HSYA.

### 3.4. Animals

Adult Wistar rats of either sex (150–200 g) were used in the studies accredited by the Institutional Animal Care and Use Committee (IACUC) of Harbin Medical University. The rats were housed in the Animal Research Center at a controlled ambient temperature of 22 ± 2 °C with 50 ± 10% relative humidity and a 12-h light-dark cycle (lights on at 8:00 AM). Standard rat chow and water were provided *ad libitum* to all rats. After the rats were deeply anesthetized with an intraperitoneal injection of 1% chloral hydrate according to their weight, PAs were carefully dissected from adult rats and cut into 3-mm-length rings under a dissecting microscope, maintained in cold Krebs solution containing (in mM) NaCl 127, KCl 4.7, NaHCO_3_ 17, MgSO_4_ 1.17, KH_2_PO_4_ 1.18, CaCl_2_ 2.5, and D-glucose 5.5 (pH 7.4, at 37 °C). In the case of endothelium-denuded preparations, the vessels were mechanically rubbed by a wolfram wire before equilibration. Endothelial integrity was examined in all experiments by the presence of the characteristic relaxation response to carbamyl choline chloride (5 × 10^−6^ M). After equilibration, each ring was contracted with 10^−6^ M PE followed by treatment with carbamyl choline chloride. Vascular rings displaying less than 10% relaxation to carbamyl choline chloride were considered as endothelium-denuded.

### 3.5. Tension Studies of Pulmonary Arterial (PA) Rings

Each PA ring was mounted on a ALC-MPA force transducer and placed in a water-jacketed organ bath. The system allows simultaneous recordings from eight rings. The rings were bathed with 3 mL oxygenated Krebs solution. PA rings were equilibrated under 0.3 g passive tension for 30 min. All rings were contracted with 1.0 mmol/L phenylephrine (PE) to ensure tissue vitality at the end of the experiment. Rings which did not exhibit a doubling of tension to PE were excluded from analysis. To study the vasorelaxation properties of HSYA in the PA rings with and without endothelium, the rings were pre-incubated with HSYA (10^−9^–10^−5^ M respectively) for 40 min, then cumulative concentration-response curves to PE were produced by adding increasing concentrations of PE (10^−9^ to 10^−5^ M) at 5-min internal, and examining the effects of HSYA.

### 3.6. Statistical Analysis

The experimental data are expressed as means ± SEM. Statistical analysis was performed with one-way ANOVA followed by Dunnett’s test where appropriate. *p* < 0.05 was considered statistically significant.

## 4. Conclusions

It may be concluded that *Carthamus tinctorius* L. flower shows pulmonary vascular relaxation effects. Our studies show that HSYA, the main constituent of *Carthamus tinctorius* L. flowers, exhibits strong effects against the vascular contractile response of PE. Finally we conclude that HSYA may be a potential pulmonary vascular relaxant for the treatment of pulmonary hypertension diseases like PAH, and it’s underlying mechanism is independent of PA endothelium, and it contributes to the function of pulmonary vascular smooth muscles (PVSMs), HSYA reduces the vascular tension through activating the Kv channel of PVSMCs, so it may be a potential medication for PAH.
